# Five Layers of Receptor Signaling in γδ T-Cell Differentiation and Activation

**DOI:** 10.3389/fimmu.2015.00015

**Published:** 2015-01-26

**Authors:** Sérgio T. Ribeiro, Julie C. Ribot, Bruno Silva-Santos

**Affiliations:** ^1^Faculdade de Medicina, Instituto de Medicina Molecular, Universidade de Lisboa, Lisboa, Portugal

**Keywords:** γδ T-cells, T-cell receptor, T-cell costimulation, cytokines, natural killer receptors

## Abstract

The contributions of γδ T-cells to immunity to infection or tumors critically depend on their activation and differentiation into effectors capable of secreting cytokines and killing infected or transformed cells. These processes are molecularly controlled by surface receptors that capture key extracellular cues and convey downstream intracellular signals that regulate γδ T-cell physiology. The understanding of how environmental signals are integrated by γδ T-cells is critical for their manipulation in clinical settings. Here, we discuss how different classes of surface receptors impact on human and murine γδ T-cell differentiation, activation, and expansion. In particular, we review the role of five receptor types: the T-cell receptor (TCR), costimulatory receptors, cytokine receptors, NK receptors, and inhibitory receptors. Some of the key players are the costimulatory receptors CD27 and CD28, which differentially impact on pro-inflammatory subsets of γδ T-cells; the cytokine receptors IL-2R, IL-7R, and IL-15R, which drive functional differentiation and expansion of γδ T-cells; the NK receptor NKG2D and its contribution to γδ T-cell cytotoxicity; and the inhibitory receptors PD-1 and BTLA that control γδ T-cell homeostasis. We discuss these and other receptors in the context of a five-step model of receptor signaling in γδ T-cell differentiation and activation, and discuss its implications for the manipulation of γδ T-cells in immunotherapy.

## Introduction

γδ cells endow the T-cell compartment with a rapid, innate-like reaction to insults, which places them in the afferent phase of the immune response. Namely, γδ T-cells are responsible for “lymphoid stress surveillance,” i.e., sensing and responding immediately to infections or non-microbial stress without the need of clonal expansion or *de novo* differentiation, in synchrony with prototypic innate immune responses (1). Critically, this implicates γδ T-cells in inflammation (2), autoimmunity (3), infectious diseases (4, 5), and tumor surveillance (6–8).

Many of the studies elucidating the physiological roles of γδ T-cells have been performed in murine models, where a major breakthrough has been the identification of pro-inflammatory subsets naturally producing either IFNγ or IL-17 (9–11). Moreover, these studies have been greatly facilitated by the identification of cell surface markers that segregate the two functional γδ T-cell subsets: CD27, CD122, and NK1.1 mark IFNγ-producing γδ cells, whereas their IL-17-expressing counterparts display a CD27^−^ CCR6^+^ phenotype (9–11). Moreover, the two subsets show distinct Vγ chain usage in their TCR repertoires, with a bias toward Vγ1 among IFNγ-producing γδ cells, and an enrichment in Vγ4 and Vγ6 in IL-17-producing γδ cells (12).

In humans, γδ T-cells are primarily identified by their Vδ chain usage, with Vδ1^+^ cells predominating in the thymus and in peripheral tissues, while Vδ2^+^ cells (mostly co-expressing a Vγ9 chain) constitute the majority of blood-circulating γδ T-cells. Both human γδ T-cell subsets are highly prone to secrete IFNγ, but IL-17 can be induced in highly inflammatory conditions triggered by infections (13) or tumors (14, 15).

In both murine and human γδ T-cells, functional responses are initiated upon recognition of antigens that are likely induced by stress signals and sensed by either T-cell or natural killer receptors. Some γδ T-cell populations are also particularly responsive to cytokines or innate toll-like receptor (TLR) agonists (16, 17). Following proliferation and effector responses, the return to homeostasis is controlled by inhibitory receptors. Here, we discuss the various layers of contributions of T (TCR and costimulatory/inhibitory receptors), NK, and cytokine receptors to the activation and differentiation of effector γδ T-cell populations in mice and humans.

## Signal 1: T-Cell Receptor

The γδTCR complex is composed by the γδTCR itself and various CD3 chains following the stoichiometry: TCRγδCD3δ_2_γδζ_2_ in humans and TCRγδCD3δ_2_γ_2_ζ_2_ in mice (18). The assembly of a γδTCR complex in thymic progenitors has immediate consequences for γδ T-cell development. The “strong” signals stemming from the γδTCR (when compared to the “weaker” pre-TCR signaling) drive γδ/αβ common precursors into the γδ lineage (19, 20). These “stronger” γδTCR signals associate with increased phosphorylation of ERK1/2, abundant calcium release and induction of early growth response (Egr) transcription factors (21, 22).

The TCR complex does not present intrinsic kinase activity but the intracellular signaling is initiated after phosphorylation of immunoreceptor tyrosine-based activation motifs (ITAMs) in the CD3 cytoplasmic domains by the Src-family kinases (SFKs) Lck and Fyn (23). The recruitment of these SFKs to the TCR complex in γδ T-cells remains obscure since these cells do not express the CD4 or CD8 co-receptors, which have been shown, in αβ T-cells, to be responsible for recruiting SFKs upon αβTCR ligation (23). Nonetheless, the importance of SFKs in γδ T-cells is underscored by the substantial phosphorylation of ERK upon inhibition of Csk, a potent inhibitor of SFKs (24).

SFK-mediated phosphorylation of the ITAMs on CD3 chains allows the recruitment, phospholylation, and activation of Zap70 that facilitates phosphorylation of the scaffolding proteins SLP-76 and LAT. This lead to the formation of a supramolecular signalosome that recruits the phospholipase PLCγ1, resulting in propagation of downstream signaling events (22). Here again, γδ T-cell signaling is different from αβ T-cells, since mutations on the binding site of PLCγ1 on LAT resulted in a severe block in murine αβ thymocyte development while γδ T-cell numbers were only modestly reduced in the thymus, intestine, and liver, and remained normal in the skin. Unexpectedly, a population of γδ T-cells in the secondary lymphoid organs in these mice underwent uncontrolled expansion and caused autoimmune pathology, suggesting distinct functions for LAT/PLCγ1-mediated signaling in subpopulations of γδ T-cells (21, 25).

In humans, the major γδ T-cell subset in the peripheral blood, Vγ9Vδ2 T-cells, are uniquely and specifically reactive to self- and foreign non-peptidic phosphorylated intermediates of isoprenoid synthesis – “phosphoantigens” or “phosphoagonists” (P-Ags) (26–28). These P-Ags were shown to trigger bona fide Vγ9Vδ2 TCR signaling in various studies. Cipriani and colleagues showed that the activation of Vγ9Vδ2 T-cells with the P-Ag isopentenyl pyrophosphate (IPP), induced rapid and persistent PKC-dependent phosphorylation of ERK1/2, p38 MAPK, and JNK, resulting in NF-κB and AP-1 activation as well as the release of MIP-1α, MIP-1β, IFN-γ, and TNF-α (29). Moreover, P-Ag stimulation and CD3-crosslinking produced identical phosphorylation of the signaling proteins Zap70, PI3K, LAT, ERK1/2, and p38 MAPK (30, 31); and induced highly sustained calcium signaling in Vγ9Vδ2 T-cells (32). Importantly, activation by P-Ags is the basis of current cancer immunotherapy strategies involving Vγ9Vδ2 T-cells (33).

Recent work has produced some puzzling results on the role of the γδTCR in the development of effector subsets of murine γδ T-cells (34–36), namely, CD27^+^ CD122^+^ γδ T-cells producing IFN-γ or CD27^−^ CCR6^+^ γδ T-cells making IL-17 (9, 10). First, Chien and co-workers showed that T10/T22-specific γδ T-cells required thymic expression of their TCR ligand to differentiate into IFN-γ producers, in contrast with “ligand naïve” IL-17 producers (9). Consistent with this, TCR-dependent thymic selection was also shown to set the functional potential of dendritic epidermal T-cells (DETC) progenitors away from IL-17 production (37). Furthermore, peripheral IL-17-producing CD27^−^ CCR6^+^ γδ T-cells were shown to expand and produce IL-17 independently of TCR activation (38). However, a subsequent study by Chien and collaborators demonstrated that a subset of phycoerythrin (PE)-specific γδ T-cells produced IL-17 specifically upon TCR ligation (39). Moreover, a recent study by Hayday and colleagues suggested that an impairment in Zap70 signaling (in SKG mice) mostly affected the development of IL-17^+^ rather than IFN-γ^+^ γδ T-cells (40). The authors further proposed that “innate-like” γδ T-cell populations, including IL-17 producers and some subsets of IFN-γ producers, receive strong TCR signals during thymic development to become hyporesponsive to TCR stimulation in the periphery (40). Future research should aim to resolve the apparent contradictions of the available data, namely, by clarifying the requirement on TCR ligand engagement, as well as the developmental effects of manipulating distinct γδTCR signaling pathways and their downstream (transcriptional and post-transcriptional) mechanisms on γδ T-cell subsets.

## Signal 2: Costimulatory Receptors

A series of T-cell costimulatory receptors are known to induce qualitative and quantitative changes that lower activation thresholds, prevent “anergy” and enhance T-cell functions. Typical costimulatory receptors are type I transmembrane proteins that can be divided into two groups, based on their structural characteristics: immunoglobulin (Ig) or tumor necrosis factor receptor (TNFR) superfamilies. Ig superfamily members have a variable Ig-like extracellular domain and a short cytoplasmic tail, whereas TNFR family members present extracellular domains rich in six cysteine repeats (which form disulfide bridges) and a more complex cytoplasmic tail [reviewed in Ref. (41)]. These two main types of costimulatory receptors display different modes of intracellular signaling: whereas the CD28 family members associate directly with protein kinases (like PI3K or ITK), TNFR superfamily co-receptors require the adaptor proteins TRAF (TNFR-associated factor), namely TRAF2 and TRAF5, to link to downstream signaling mediators (Table 1). Here, based on their specific roles in γδ T-cells, we shall discuss CD28 (of the Ig superfamily) and the TNFR superfamily members, CD27, CD30, and CD137 (4-1BB).

**Table 1:** **Co-receptors of γδ T-cells – extracellular ligands and intracellular signaling pathways**.

Receptor	Ligands	Intracellular signaling initiators/adaptors	Downstream signaling pathway	Target molecules	Reference
CD28	B7.1 (CD80) B7.2 (CD86)	PI3K ITK Grb2	PI3K/AKT Grb2/MEK/ERK	IL-2, NF-κB, AP-1, Bcl-x_L_, NFAT	(42–45)
CD27	CD70	TRAF2 TRAF5 Siva	IKK/NF-κB JNK	NF-kB, Ca^2+^, *cyclinD2*, *Bcl2a1*, Bcl-x_L_	(46–49)
CD30	CD30L	TRAF2 TRAF5	TRAF/IKK/IkB Ca^2+^	NF-κB, IL-4, IFNγ, IL-8, CC chemokines	(46, 50, 51)
4-1BB (CD137)	CD137L	TRAF2		NF-κB, IFNγ	(52 –54)
IL-2R IL-15R	IL-2 IL-15	Jak1 Jak3	PI3K/AKT Jak/STAT4/STAT5 MEK/ERK STAT1	IFNγ, TNF-α, T-bet, eomesodermin	(55–58)
IL-7R	IL-7	Jak1 Jak3	STAT3	IL-17, SOCS3	(59)
IL-21R	IL-21	Jak1 Jak3	STAT3	CXCL13, CXCR5	(60)
NKG2D	MIC(A–B)	DAP10	PI3K/AKT	NF-κB, RelB, Bcl-x_L_, Bcl-2	(32, 46, 61–63)
	ULBP (1–6)		Grb2/VAV1/SOS1	
	H60		PKCθ/Ca^2+^	
	MULT1	
	RAE1	
NKp30	B7-H6 BAT3	CD3ζ	cAMP/PKA	CC chemokines: CCL3, CCL4, CCL5	(64–67)
NKp44	NKp44L	DAP12	Zap70/Syk		(64, 68–70)
DNAM-1 (CD226)	Nectin-like-5 Nectin-2	PKC LFA-1 Fyn	SLP-76/VAV1/ERK		(71, 72)
PD-1	PD-L1 (B7-H1) PD-L2 (B7-DC)	SHP-1 SHP-2	CK2/PTEN/PI3K/AKT MEK/ERK	GSK-3, Bcl-x_L_ Smad3, Cdc25A, IFNγ, IL-2	(73–76)
BTLA	HVEM	SHP-1	Zap70/ERK	IL-17, TNF, IL-2	(77–79)
		SHP-2	

The best studied costimulatory receptor, CD28, has historically yielded paradoxical results on γδ T-cells (46). We have recently readdressed this issue for both human and mouse γδ T-cells. We described that CD28 is constitutively expressed on lymphoid γδ T-cells and promotes survival and proliferation via IL-2 production. CD28 receptor agonists enhanced γδ T-cell expansion, which was conversely inhibited by blocking antibodies against its B7 ligands (42). Importantly, CD28-deficient mice displayed lower (relative to controls) numbers of total or activated γδ T-cells upon *Plasmodium berghei* infection, and failed to expand both their IFN-γ^+^ and IL-17^+^ subsets (42). In contrast, Hayes and colleagues reported that both functional γδ T-cell subsets differentiated and expanded normally in a *Listeria* model (80). It would be interesting to determine how variable is the dependence on CD28 costimulation for γδ T-cell responses to distinct infectious agents.

In naïve mice, while CD28 is not required for the development of either IFN-γ^+^ or IL-17^+^ γδ T-cell subsets (80), the TNFR superfamily member CD27 is selectively implicated in the generation of IFN-γ^+^ γδ T-cells (10). In fact, we showed that CD27 expression segregates IFN-γ^+^ (CD27^+^) and IL-17^+^ (CD27^−^) γδ T-cells. Most interestingly, these phenotypes are established in the thymus, and since embryonic stages. Based on the results from our (10) and Chien’s (9) teams, the development of IFN-γ-producing γδ T-cells seemingly requires strong TCR signaling and CD27 costimulation in the thymus.

Beyond its role in thymic differentiation, CD27 is critical for the expansion of peripheral IFN-γ-producing γδ T-cells upon infection with herpes viruses or malaria parasites in mice (81). We showed that, in the context of TCR stimulation and upon ligation to CD70, CD27 signaling activates the non-canonical NF-κB pathway and enhances the expression of anti-apoptotic and cell cycle-related genes, thus promoting murine γδ T-cell survival and proliferation (81).

We have also addressed the impact of CD27 costimulation on the activation of human γδ T-cells. Administration of soluble recombinant CD70 enhanced, whereas anti-CD27 (or anti-CD70) antibodies reduced, Vγ9Vδ2 T-cell expansion *in vitro* (82). Moreover, CD27 signals induced calcium fluxes and upregulated the expression of *Cyclin D2* and the anti-apoptotic gene *Bcl2a1*. Given the typical IFN-γ secretion and cytotoxicity of activated Vγ9Vδ2 T-cells (30), our work suggests that the modulation of CD70–CD27 signals may be beneficial in the context of γδ T-cell-based cancer immunotherapy.

Upon activation, human γδ T-cells can also express another TNFR superfamily member, CD30 (83). CD30 signaling, which potentiated calcium fluxes induced by TCR activation, also enhanced pro-inflammatory cytokine production (50). Recently, Yoshikai and colleagues compared γδ T-cell homeostasis and response to *Listeria monocytogenes* in CD30-sufficient versus deficient mice. They demonstrated a selective depletion of IL-17-producing Vγ6^+^ T-cells in mucosal tissues in the steady-state and upon infection (84). This associated with reduced bacterial clearance, which could be rescued, alongside the IL-17^+^ Vγ6^+^ T-cell pool, by agonistic anti-CD30 antibody administration. In contrast, Lee et al. reported that agonistic anti-CD137 (4-1BB) antibodies promoted the expansion of IFN-γ^+^ Vγ1^+^ T-cells, which protected (in an IFN-γ-dependent manner) also from *Listeria* infection (52). This study also showed that 4-1BB was expressed and functional on activated human γδ T-cells, and its ligation upon cell transfer protected NOD/SCID mice against *Listeria* infection.

Interestingly, activated Vγ9Vδ2 T-cells also express high levels of 4-1BBL (CD137L) (85), which besides acting as a ligand for 4-1BB on T and NK-cells, may also participate in Vγ9Vδ2 T-cell activation due to its known reverse signaling ability (86). This may, in fact, also apply to CD70 (CD27-ligand), which is highly induced upon phosphoantigen-mediated stimulation of Vγ9Vδ2 T-cells (82, 87). These possibilities deserve further investigation.

## Signal 3: Cytokine Receptors

Interleukins are key determinants of T-cell survival, proliferation, and differentiation. IL-7, IL-15, and IL-2 are essential for lymphocyte development and homeostasis; upon inflammation, other cytokines, namely, IL-1β, IL-12, IL-18, IL-21, and IL-23, take a central role in determining T-cell functions. Here, we review the main contributions of homeostatic and inflammatory cytokines specifically to γδ T-cell physiology.

IL-7 and IL-15 are seemingly the key determinants of murine γδ T-cell development (88–90) and homeostasis (91). A recent study that depleted IL-7 specifically from (Foxn1^+^) thymic epithelial cells showed that γδ T-cells were significantly reduced in the adult thymus and in the gut, whereas they were completely absent in the fetal thymus and epidermis (89). In the dermis, it was also IL-7, but not IL-15, that supported the development and survival of the resident γδ T-cell population (92). Conversely, in the gut, IL-15 seems to play the primordial role in sustaining the local intraepithelial γδ T-cell compartment (93).

Unexpectedly, IL-7 was recently reported to promote the selective expansion of murine IL-17-producing γδ T-cells (59). STAT3-dependent IL-7 signals allowed CD27^−^ γδ T-cells to resist activation-induced cell death (AICD) and undergo proliferative responses to TCR agonists. Such an IL-7/IL-17 axis was also reported to be required for the γδ T-cell response to viral hepatitis infection *in vivo* (94). Moreover, IL-7 also seems to support the expansion of human IL-17-producing γδ T-cells (59).

We recently assessed the functional differentiation of human γδ thymocytes, which are >80% of the Vδ1 subtype. We observed that IL-15 and IL-2, but not IL-7, induced the cytotoxic type 1 (IFN-γ-producing) program in functionally immature γδ thymocytes (55). This was consistent with previous data on peripheral γδ T-cells isolated from cancer patients (95). However, additional reports on peripheral Vγ9Vδ2 T-cell cultures showed that IL-15 or IL-2 stimulation, despite efficient ERK and AKT activation, were not sufficient to induce effector responses; these required phosphoantigen-dependent TCR activation and downstream calcium mobilization (56, 96). Unexpectedly, in our cultures of γδ (mostly Vδ1) thymocytes, TCR stimulation was not required for neither ERK activation nor T-bet and eomesodermin induction and the acquisition of effector functions (55).

IL-2 and IL-15 play key roles in the peripheral expansion of Vγ9Vδ2 T-cells in response to microbial phosphoantigens or synthetic drugs like bisphosphonates (56, 97). This notwithstanding, it is important to note, toward the therapeutic application of Vγ9Vδ2 T-cells, that optimal effector responses seemingly require the combination of these cytokines with TCR agonists. Thus, recent work from Chen and colleagues demonstrated that the differentiation of cytotoxic type 1 Vγ9Vδ2 T-cells capable of controlling *Mycobacterium tuberculosis* infection in macaques required a phosphoantigen/IL-2 combination (98).

Effector γδ T-cell differentiation is also greatly impacted by inflammatory cytokines, particularly IL-12 and IL-18 that typically promote IFN-γ production; and IL-1β and IL-23 that mostly drive IL-17 production.

High expression of IL-12Rβ expression on activated murine γδ T-cells guarantees a dominance of type 1 (IFN-γ^+^) over type 2 (IL-4^+^) effector fates (99). Type 1 differentiation is also predominant in human γδ T-cells, and can be further enhanced by IL-18 (100, 101) or IL-21 (102). The induction of a type 17 program in human γδ T-cells requires persistent stimulation with IL-23 for neonatal Vγ9Vδ2 T-cells (15); and IL-23 and IL-1β in the presence of TGF-β for adult Vγ9Vδ2 T-cells (13, 103). In mice, IL-1β and IL-23 are also the main drivers of abundant IL-17 production by peripheral γδ T-cells (3, 5, 81, 104–106), although recent data surprisingly suggest that IL-18 can replace IL-1β in combining with IL-23 to induce IL-17 expression (107). In contrast, IL-1β upstream of IL-1R seems essential for GM-CSF production by γδ T-cells (108).

Finally, IL-21 was recently suggested to endow human Vγ9Vδ2 T-cells with B-cell helper activity associated with a T follicular helper cell-like phenotype (60, 109), which may impact on the generation of high affinity antibodies against microbial infections.

## Signal 4: Natural Killer Receptors

An important key characteristic that allows the recognition of transformed cells by γδ T-cells is the expression of a wide set of germline-encoded receptors that were initially described in NK-cells and hence are collectively known as NK receptors (NKRs), including natural cytotoxicity receptors (NCRs).

The C-type lectin-like NK receptor group 2 member D (NKG2D) is the best studied NKR in γδ T-cells. NKG2D binds extracellularly to multiple ligands of the MIC(A–B) and ULBP (1–6) families in humans; and to H60, MULT1, and various RAE1 molecules in mice (110). NKG2D ligands are induced upon cellular stress, for example, downstream of the DNA-damage response pathway in tumor cells (111, 112). The biological significance of this recognition system is underlined by the increased susceptibility of NKG2D-deficient mice to tumor development (113).

Intracellularly, NKG2D binds to DNAX-activating protein of 10 kDa (DAP10), which carries an YXNM motif that after tyrosine phosphorylation recruits PI3K or a Grb2–Vav1–SOS1 signaling complex (Table 1). This motif is similar to that in CD28, and thus, NKG2D/DAP10 may provide T-cells with costimulatory signals that synergize with the ITAM-based TCR/CD3 complex (61). However, unlike αβ T-cells but similarly to NK-cells, γδ T-cells can express both DAP10 and DAP12 (62). The latter contains an ITAM motif, which after tyrosine phosphorylation recruits and activates Syk and ZAP70. Interestingly, only murine but not human NKG2D is able to associate with DAP12 (in addition to DAP10).

The controversy on a primary stimulatory versus costimulatory role of NKG2D in γδ T-cells has been discussed elsewhere (46, 114). Briefly, the costimulatory function of NKG2D in human Vγ9Vδ2 T-cells was supported by additive effects on TCR-mediated activation: an upregulation of cytokine production upon MICA-NKG2D interactions (115); and an increase in intracellular calcium mobilization and cytotoxic activity (32). However, other lines of evidence have suggested that NKG2D signals can activate γδ T-cells in the absence of TCR engagement: NKG2D ligation can upregulate CD69 expression in Vγ9Vδ2 T-cells to similar extent as TCR stimulation (116); NKG2D but not TCR blockade can inhibit Vγ9Vδ2 T-cell cytotoxicity against various hematological tumors (117); and murine DETC can target tumors upon recognition of NKG2D ligands (6, 118).

Another NKR implicated in tumor cell recognition by Vγ9Vδ2 T-cells is DNAX accessory molecule-1 (DNAM-1). DNAM-1 is an Ig-like family glycoprotein composed of a cytoplasmic domain containing three putative sites of phosphorylation by intracellular kinases. The phosphorylation of the Ser329 by protein kinase C (PKC) was shown to be critical for the association between DNAM-1 and LFA-1, which recruits the Fyn Src kinase to phosphorylate the Tyr322 of DNAM-1, thus initiating downstream signaling leading to SLP-76 and Vav1 phosphorylation (Table 1) (119). Antibody-mediated DNAM-1 blockade impaired Vγ9Vδ2 T-cell cytotoxicity and IFN-γ production against hepatocellular carcinoma lines expressing Nectin-like-5 (71).

Recently, we characterized a Vδ1^+^ T-cell population capable of targeting hematological tumors resistant to fully activated Vγ9Vδ2 T-cells (120). Unexpectedly, the enhanced killer function resulted from induced NCR expression, namely NKp30 and NKp44, which had been previously regarded as NK-specific markers. Although neither Vδ1^+^ nor Vδ2^+^ cells express NCRs constitutively, these can be upregulated selectively in Vδ1^+^ cells by PI3K/AKT-dependent signals provided by γc cytokines (IL-2 or IL-15) and TCR stimulation. Once expressed on the cell surface, NKp30 and NKp44 can signal via CD3ζ and DAP12, respectively (64). We further showed that NKp30 and NKp44 are both functional in NCR^+^ Vδ1^+^ T-cells and synergize with NKG2D to target lymphocytic leukemia cells (120).

In sum, NKRs seem critical for tumor recognition and deployment of the cytotoxic program that is endowed by TCR/γc cytokine-dependent differentiation, thus defining distinct mechanisms to be integrated in γδ T-cell-mediated cancer immunotherapy.

## Signal 5: Inhibitory Receptors

Beyond efficient activation and deployment of effector functions, it is necessary to negatively regulate the T-cell response in order to return to the homeostatic baseline. Inhibitory receptors like PD-1 or CTLA-4 are known to be critical for this contracting phase of the T-cell response and have become major clinical targets in cancer immunotherapy. Although γδ T-cells rarely express CTLA-4, they can upregulate PD-1 upon activation, while they constitutively express BTLA, and thus these two receptors may be the key to control γδ T-cell responses.

Programed death-1 (PD-1) is absent or low expressed on circulating Vγ9Vδ2 T-cells but is rapidly induced upon activation (121). The cytoplasmic tail of PD-1 contains conserved immunoreceptor tyrosine-based inhibitory motif (ITIM) and switch motif (ITSM), both of which are phosphorylated to recruit negative regulators that block Lck activity downstream of the TCR complex (122). Moreover, PD-1 ligation can augment the activity of the protein phosphatase and tensin homolog (PTEN), a cellular phosphatase that inhibits PI3K/AKT signaling and thus leads to impaired survival, proliferation, and IL-2 release (123). The expression of the ligand PD-L1 on tumor cells inhibited Vγ9Vδ2 T-cell cytotoxicity and IFN-γ production (121). However, zoledronate-induced accumulation of P-Ags in tumor cells and consequent Vγ9Vδ2 TCR activation seemed to overcome the inhibitory effect of PD-1/PD-L1 interactions. More research is required to understand the full extent to what PD-1 may control γδ T-cell functions and homeostasis.

B- and T-lymphocyte attenuator (BTLA) is another inhibitory receptor, member of the CD28 family and structurally related to PD-1 and CTLA-4. Binding to its ligand, herpesvirus entry mediator (HVEM), induces phosphorylation of the ITIM domain and association with SH2 domain-containing protein tyrosine phosphatase 1 (SHP-1) and SHP-2, which leads to attenuation of cellular activation and growth (124). Recent data showed that BTLA engagement with HVEM reduced P-Ag/TCR-mediated signaling and inhibited Vγ9Vδ2 T-cell proliferation, including in response to lymphoma cells (77). Conversely, BTLA-HVEM blockade using monoclonal antibodies enhanced Vγ9Vδ2 TCR signaling and may thus have therapeutic potential for the positive manipulation of γδ T-cells.

A detailed study on BTLA function in murine γδ T-cells has revealed a selective involvement in the homeostasis of the IL-17-producing CD27^−^ γδ T-cell subset (78). Although these cells constitutively express low levels of BTLA, it is upregulated by IL-7 stimulation and thereby limits γδ T-cell numbers. Consequently, BTLA-deficient mice accumulated IL-17^+^ CD27^−^ γδ T-cells and were more susceptible (than wild-type controls) to dermatitis, which could be reversed by agonist BTLA antibodies. Thus, BTLA may be an important target for controlling pathogenic γδ T-cells in inflammatory and autoimmune diseases.

## Concluding Remarks

A multitude of surface receptors has been shown to participate in γδ T-cell differentiation and activation. However, some crucial aspects remain to be elucidated, such as the identity of most γδTCR ligands. Most importantly, we must improve the transfer of past and current basic research into future protocols for γδ T-cell-based immunotherapy. In this context, some key questions are: how to balance γδTCR activation with “exhaustion” due to chronic stimulation? What can be achieved by manipulating the NK-like activation mode of γδ T-cells? Which costimulatory receptors should be modulated, and at what stages, to boost the desired γδ T-cell responses? Which combinations of cytokines enable the best effector γδ T-cells for each therapeutic application? Which receptors are most useful to tune down or switch off pathogenic effector γδ T-cells? The answers to these questions must be obtained in appropriate *in vivo* pre-clinical models and hopefully next in the clinic.

For now, we would like to propose that the five types of receptor signals reviewed here define five distinct layers of regulation of γδ T-cell differentiation, activation, and function. The γδTCR is critical for the initial stages of differentiation and for proliferative responses; both processes further require cytokine signals that promote cell survival, proliferation, and terminal effector function. Costimulatory and inhibitory receptors control the extent of γδ T-cell expansion, with interesting biases toward specific effector subsets. Finally, NK receptors play a decisive role in tumor cell targeting by γδ T-cells. Thus, we believe that the recognition of “stressed self” can be mediated by the γδTCR but also chiefly by NK receptors like NKG2D. As such, the characterization of both type of ligands on tumors may be critical to design protocols, select and monitor patients, and increase the chances of efficacious γδ T-cell-based cancer immunotherapies.

## Conflict of Interest Statement

Bruno Silva-Santos is a co-founder and share holder of LymphAct S.A. The other co-authors declare that the research was conducted in the absence of any commercial or financial relationships that could be construed as a potential conflict of interest.
